# Osteogenesis imperfecta in children: Indosesian experiences

**DOI:** 10.1186/1687-9856-2013-S1-P166

**Published:** 2013-10-03

**Authors:** Nur Rochmah, Nanis S Marzuki, Eka Agustiarini, Aman B Pulungan

**Affiliations:** 1 Department of Pediatrics, School of Medicine, Airlangga University, dr Soetomo Hospital, Surabaya, Indonesia; 2 Eijkman Institute for Molecular Biology, Jakarta, Indonesia; 3 Department of Pediatrics, School of Medicine, Andalas University, Muhammad Djamil Hospital, Padang, Indonesia; 4 Department of Child Health, Faculty of Medicine, University of Indonesia, Ciptomangunkusumo Hospital, Jakarta, Indonesia

## 

Medical management of osteogenesis imperfecta (OI), a genetic disorder of connective tissue characterized by reduced bone mass and frequent fractures, is focused on maximizing patients’ mobility and function. Many methods of pharmacological treatment have been introduced for the severe forms of OI, including calcium, anabolic steroids, growth hormone, and calcitonin, without consistent benefit. Recent studies reported beneficial effect of intravenous and oral bisphosphonates on moderate to severe form of OI in children.

This report is to review OI cases in Indonesian children and to know the effect of bisphosphonate treatment for them.

Data on age, gender, age at diagnosis, availability of bisphosphonate treatment, and its effects were collected from Indonesian OI children registry, which documented from April to June 2012. Fourteen centers which have members of pediatric endocrinology chapter, Indonesian Pediatric society, were involved in obtaining data for the registry.

Forty-six OI cases (17 female), who were diagnosed at the age of 7 days-11 years old and mostly categorized as type 3 of OI, were recorded. Two patients were died before commencing treatment. There were twenty-seven (27/46) patients received bisphosphonates, which were administered to moderate to severe cases. Two patients,who were treated, were died due to hyperthermia after the first day of pamidronate infusion, and pneumonia aspiration. Twenty-five patients received intravenous pamidronate and 3 oral alendronate. Liver and renal function test, and serum electrolyte were evaluated before and after treatment. Most patients reported decreased irritability, increased muscle tone and mobility. In addition, despite hyperthermia in one case, no serious adverse effects documented. Although some patients had government health insurance for the poor, unfortunately, the medicine, which is considered expensive for the patients, is not yet included in the drug list of government insurance.

Most of registered OI cases in Indonesian children were type 3. More than half of the cases underwent treatment with bisphosphonates with beneficial effects, although long-term follow up and well-documented data are required.

